# End-tidal CO corrected for ambient CO risk adjusted phototherapy threshold for the management of neonatal hyperbilirubinemia: a randomized clinical trial

**DOI:** 10.1007/s12519-025-00954-y

**Published:** 2025-08-04

**Authors:** Ge Yang, Li Deng, Kun Zhang, Hui-Juan Liu, Xin-Rui Fu, Yue Hu, Xiao-Dan Yan, Xiao-Yun Zhou, Wei Luo, Si-Yao Wang, Xiao-Tong Ye, Tian-Lang Zhang, Fan Li, Zhuan-Xia Huo, Yan Jiang, Shan Zeng, De-Hua Wu, Yuan Yuan, Hua-Yan Zhang

**Affiliations:** 1 Division of Neonatology and Center for Newborn Care, Guangzhou Women and Children’s Medical Center, Guangzhou Medical University, No. 9 Jinsui Road, Tianhe District, Guangzhou, 510623 Guangdong Province People’s Republic of China; 2 Division of Neonatology, Department of Pediatrics, The Children’s Hospital of Philadelphia and the University of Pennsylvania Perelman School of Medicine, Philadelphia, PA USA; 3 Clinical Research Center, Guangzhou Women and Children’s Medical Center, Guangzhou Medical University, Guangzhou, China

**Keywords:** End-tidal carbon monoxide, Neonatal hyperbilirubinemia, Phototherapy, Randomized clinical trial

## Abstract

**Background:**

Neonatal hyperbilirubinemia risk factors determination is challenging due to the lack of quantifiable indicators for bilirubin production, resulting in phototherapy decisions made without real-time information. End-tidal carbon monoxide (CO) corrected for ambient CO (ETCOc) may be helpful for identifying hemolysis, but evidence on the application of ETCOc as a risk factor for the development of neonatal hyperbilirubinemia is scarce. This study aimed to evaluate whether the use of ETCOc to adjust neonatal hyperbilirubinemia risk categories and thus phototherapy thresholds can reduce the rate of phototherapy within the first seven days of life.

**Methods:**

This is a randomized clinical trial including near-term and term infants with a transcutaneous bilirubin $>$ 40th percentile within 72 hours after birth in a single center in Guangdong, China. Newborns were randomized to receive ETCOc-adjusted risk assessment or empirical assessment per local practice to establish phototherapy thresholds. The primary outcome was the rate of phototherapy within seven days of life. Secondary outcomes were postnatal hours at phototherapy, total serum bilirubin and ETCOc before phototherapy, severe hyperbilirubinemia and phototherapy duration.

**Results:**

A total of 2500 infants were enrolled and randomized. Phototherapy within seven days of life occurred in 237 infants (18.9%) in the intervention group and 281 infants (22.5%) in the control group [adjusted relative risk: 0.85; 95% confidence interval (CI): 0.73, 0.98]. The ETCOc before phototherapy was 0.28 parts per million higher (95% CI: 0.10, 0.46) in the intervention group. The rate of subsequent severe hyperbilirubinemia was not significantly different, and other secondary outcomes were comparable between the two groups.

**Conclusions:**

For near-term and term infants at risk of neonatal hyperbilirubinemia, the use of ETCOc to adjust neonatal hyperbilirubinemia risk categories can decrease the rate of phototherapy at seven days of life. Integrating the ETCOc to adjust the phototherapy threshold is helpful in the management of severe hyperbilirubinemia.

**Graphical Abstract:**

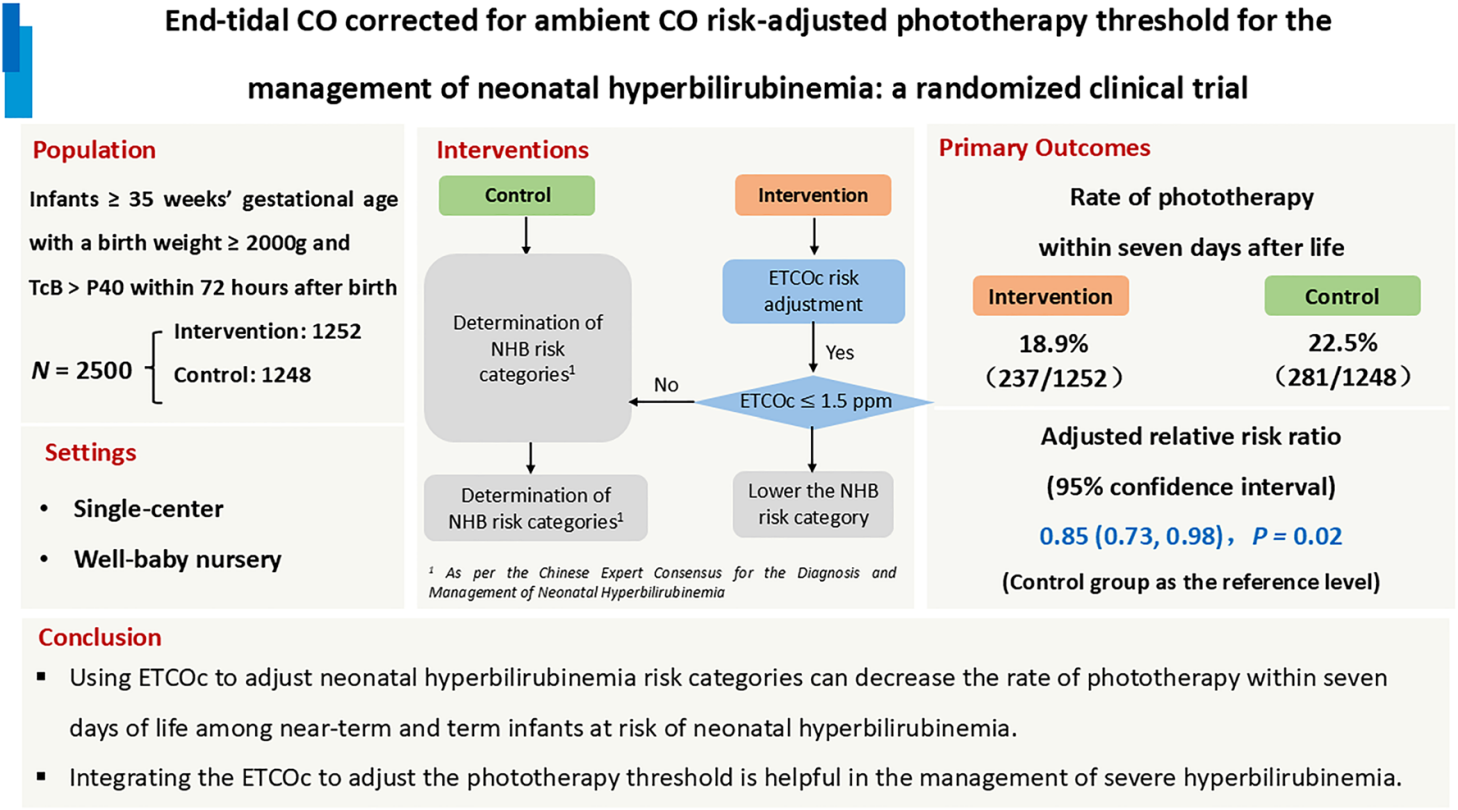

**Supplementary Information:**

The online version contains supplementary material available at 10.1007/s12519-025-00954-y.

## Introduction

Neonatal hyperbilirubinemia (NHB) is common among healthy neonates during the first 7–14 days of life [1] and remains one of the leading causes of neonatal mortality worldwide [2]. Phototherapy (PT) has been the standard of care of the NHB for decades. Over the years, the trend of PT use has reached 27%, while the incidence of jaundice has remained stable. This indicates increasing health care utilization and hospital costs related to NHB [3, 4]. NHB management relies on the assessment of risk factors for significant hyperbilirubinemia and bilirubin neurotoxicity [5, 6]. Nevertheless, determining NHB risk factors can be challenging because of the lack of recognized quantifiable indicators for bilirubin production. This situation often results in PT decisions being made without real-time information on bilirubin production.

End-tidal carbon monoxide (CO) corrected for ambient CO (ETCOc) is a real-time surrogate for quantifying bilirubin production and identifying hemolysis [7, 8] and can be measured by point-of-care ETCOc testing in both inpatient and outpatient settings. ETCOc is proportional to the rate of bilirubin production [9, 10] as CO is produced in equimolar amounts with bilirubin during heme catabolism. The incremental relationship between elevated ETCOc and total serum bilirubin (TSB) indicates that high ETCOc levels can help identify infants at the highest risk of hemolysis [8], with previous studies confirming the positive association between ETCOc and significant hemolysis in newborns [11–16]. An elevated ETCOc has also been reported to be linked with early initiation and a longer duration of PT [17, 18]. The American Academy of Pediatrics (AAP) guidelines and the Chinese Expert Consensus have suggested that ETCOc may be helpful in identifying hemolysis [5, 6, 19], but evidence on the application of ETCOc as a risk factor for the development of NHB is still scarce. There have been no existing trials evaluating the utility of the ETCOc to establish NHB risk categories and adjust PT thresholds.

Therefore, this trial was undertaken to investigate the effect of ETCOc on risk adjustment of NHB in near-term and term Chinese neonates. The primary hypothesis was that the use of an ETCOc-adjusted risk categorization assessment to establish PT initiation thresholds will decrease the rate of PT within the first seven days of life (DOLs). The secondary hypothesis was that this intervention would not increase the risk of developing severe hyperbilirubinemia.

## Methods

### Study design and participants

The study was a single-center, unblinded, parallel randomized controlled clinical trial. From November 3, 2021 to July 29, 2022, eligible infants born at the Guangzhou Women and Children’s Medical Center, Guangzhou Medical University, were recruited from a well-baby nursery. Entry criteria included infants who (1) were born at $\ge$ 35 weeks gestation with a birth weight $\ge$ 2000 g and (2) had a transcutaneous bilirubin (TcB) $>$ 40th percentile of the 2004 Bhutani nomogram [20] within 72 hours after birth. Exclusion criteria included infants with (1) major congenital anomalies (e.g., cardiac or lung abnormalities, lethal chromosomal defects), (2) immediate respiratory support after birth, nasal mucosa injury, choanal atresia or Pierre Robin sequence, or (3) self-reported active tobacco smoking or environmental tobacco smoking during the third trimester of pregnancy, which may affect the accuracy of the ETCOc measurement. The study protocol was approved by the institutional review board of the Guangzhou Women and Children’s Medical Center (No. 2021136A01) and registered in the Chinese Clinical Trial Registry (ChiCTR2100051476). This study followed the Consolidated Standards of Reporting Trials (CONSORT) reporting guidelines. Written parental/guardian consent was obtained from the participating neonates upon enrollment. This study was performed in accordance with the principles of the Declaration of Helsinki.

### Randomization and masking

Parents or legal guardians were approached for participation, and once parental consent was obtained, randomization was performed with permuted blocks (2, 4, 6 and 8) in a computer-generated random allocation sequence. Assignments were concealed in the computerized system, and research coordinators had access to the randomization result after enrollment. We randomly assigned infants with a 1:1 ratio to receive either the ETCOc-adjusted NHB risk assessment (intervention group) or the empirical risk assessment by pediatricians (control group). During the trial, infants were managed and followed up according to the group in which they were initially randomized. Masking group allocation and the intervention from pediatricians was not possible because infants were managed accordingly. The ETCOc of the controls was masked by the clinical staff and the parents.

### Procedures

For infants who were assigned to the intervention group, an ETCOc-adjusted risk assessment was conducted incorporating an ETCOc > 1.5 parts per million (ppm) as a quantifiable indicator of higher bilirubin production for a significant NHB. The threshold of 1.5 ppm was determined based on the clinical algorithm proposed by Bhutani et al. [8]: an ETCOc level ≤ 1.5 ppm indicates a decreased likelihood of hemolysis, and a level > 1.5 ppm suggests an increased likelihood. Thus, the NHB risk in interventions was adjusted to lower categories with an ETCOc $\le$ 1.5 ppm or remained consistent with empirically assessed categories as per guidelines [5, 21] if the ETCOc was $>$ 1.5 ppm (Supplemental Fig. 1). For neonates allocated to the control group, risk categories were assigned by pediatricians as per routine practice [5, 21]. PT thresholds were subsequently determined on the basis of established risk categories in both groups. The risk categories and the PT thresholds were established as per the Chinese Expert Consensus for the Diagnosis and Management of Neonatal Hyperbilirubinemia [11] in accordance with the 2004 AAP guidelines [5]. ETCOc was measured by trained coordinators who were not involved in making clinical decisions.

ETCOc was measured (CoSense® ETCOc monitor, Capnia, Inc., Foster City, CA, USA) within three hours with paired TcB (Drager JM-103, Drägerwerk AG & Co.) or TSB measurements. TcB and TSB, as needed, were measured as per routine practice. ETCOc was measured noninvasively by collecting 3–5 breath samples continuously via a disposable nasal cannula using an electrochemical sensor for CO and hydrogen, corrected for ambient CO levels. After discharge from the well-baby nursery, infants were followed up through in-person clinic visits or phone calls until either (1) the occurrence of PT within seven DOLs or (2) reaching the seven DOLs to determine the need for PT. After discharge, PT decisions were made by pediatricians or trained community health providers. Clinic visits included TcB/TSB measurements and clinical evaluation via the phototherapy nomogram, and phone follow-ups included a structured questionnaire assessing jaundice, feeding, urine/stool patterns, and parental concerns. Suspected cases required immediate TcB/TSB confirmation. Infants who required PT and were admitted to neonatal intensive care units (NICUs) within seven DOLs were managed per the local unit protocol. On admission, complete blood count, blood type, TSB and direct bilirubin, glucose-6-phosphate dehydrogenase (G6PD) activity ratio, and neonatal ABO hemolysis panel were ordered, and ETCOc was also measured before PT initiation.

### Outcome measures

The primary outcome was the rate of PT within seven DOLs, defined at the first occurrence of PT for NHB [5]. This primary outcome did not include readmission for PT. The secondary outcomes included postnatal hours at first PT, TSB and ETCOc when PT was needed, significant hyperbilirubinemia (as defined by TSB > 307.8 $\mu$mol/L) and the duration of PT.

### Power calculation and statistical analysis

Sample size was calculated on the basis of the primary outcome. Baseline data from our unit revealed that 24.4% of eligible neonates were admitted to the NICU for PT in 2020. To detect a 15% relative reduction in the rate of the primary outcome, equivalent to an absolute reduction of 3.7% (from 24.4% to 20.7%), the trial sample size was determined to require at least 2200 infants, assuming an overall $\alpha$ of 0.05, a 2-sided test with a power of 80% ($\beta$ = 0.2). Enrolling 1250 participants per group would allow for 15% loss to follow-up.

Intention-to-treat (ITT) analysis was performed on participants who were randomized and analyzed in the group to which they were initially assigned. Log-binomial regression and linear regressions were used for the analysis of adjusted estimates and 95% confidence intervals for the outcomes, adjusting for gestational age, birth weight, sex, and maternal blood group. Interactions with sex or maternal blood group were tested. Adjustment for multiple tests for the primary outcome was not prespecified. Missing data for the outcomes were assumed to be missing at random. The last available follow-up information on PT was considered the primary outcome for participants who were lost to follow-up. Two-sided *P* values less than 0.05 were considered significant. All analyses were performed via R studio (version 4.1.1, Posit Software, PBC, Boston, MA).

## Results

### Enrollment and study completion

**Fig. 1:**
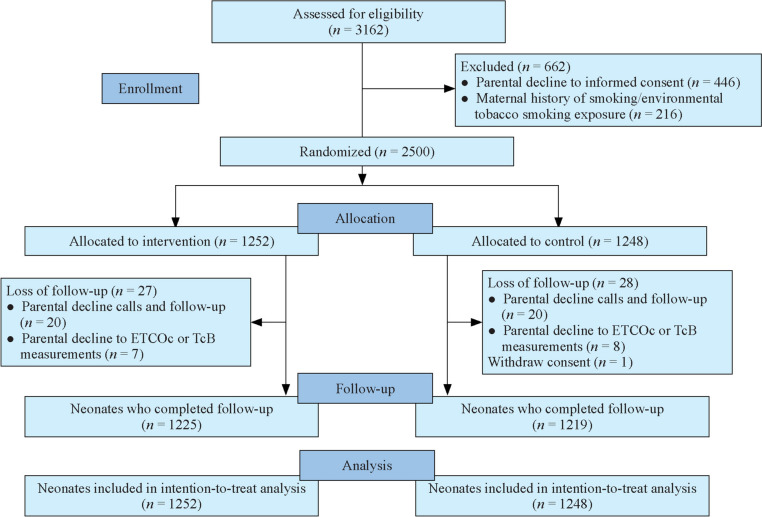
Flow chart of the study. *ETCOc* end-tidal carbon monoxide corrected for ambient CO, *TcB* transcutaneous bilirubin

During the study period, a total of 3162 neonates were screened for eligibility, and 2500 were randomized (1252 infants allocated to the intervention group and 1248 to the control group), of whom 2444 (97.8%) completed the trial (1225 in the intervention group and 1219 in the control group). Fifty-six infants did not complete the study because they were lost to follow-up (*n* = 55) and withdrew their consent after randomization (*n* = 1). As a result, 2500 infants were included in the ITT analysis (Fig. 1). The characteristics of infants who completed follow-up and those who were lost to follow-up were comparable (Supplement Table 1).

### Baseline characteristics

The trial enrolled infants with an average gestation age of 39.3 $\pm$ 1.4 weeks, and 96% were full-term neonates. A total of 1233 females and 1267 males were enrolled, with 626 females (50.2%) allocated to the control group and 607 females (48.5%) allocated to the intervention group. The two groups were comparable in terms of baseline characteristics, including gestational age, birth weight, mode of delivery, proportion of maternal ABO blood types, postnatal age at recruitment, TcB at recruitment, and number of patients lost to follow-up. The mean baseline ETCOc at recruitment was 1.68 $\pm$ 0.50 ppm in the control group and 1.71 $\pm$ 0.49 ppm in the intervention group (Table 1). Three participants had severe hyperbilirubinemia (TSB $>$ 342 μmol/L), and no acute bilirubin encephalopathy/kernicterus was diagnosed in either group during the trial. Valid readings of ETCOc were achieved in 99.2% of the ETCOc measurements.

**Table 1:** Baseline characteristics of participants

Characteristics	Participants, *n* (%)	
	Control (*n* = 1248)	Intervention (*n* = 1252)
Gestational age, median (IQR), wk	39.3 (1.4)	39.3 (1.4)
GA $<$ 37 wk, *n* (%)	47 (3.8)	48 (3.8)
GA $\ge$ 37 wk, *n* (%)	1201 (96.2)	1204 (96.2)
Birth weight, mean (SD), g	3179 (393)	3172 (393)
Female sex, *n* (%)	626 (50.2)	607 (48.5)
Mode of delivery, *n* (%)		
Vaginal delivery	688 (55.1)	687 (54.9)
Cesarean delivery	469 (37.6)	476 (38.0)
Forceps delivery	91 (7.3)	89 (7.1)
Maternal blood types, *n* (%)		
Non-type O	739 (59.2)	758 (60.5)
Type O	509 (40.8)	494 (39.5)
Postnatal age at recruitment, mean (SD), h	26.7 (13.5)	26.7 (13.8)
TcB at recruitment, median (IQR), mg/dL	6.7 (2.9)	6.8 (2.9)
ETCOc at recruitment, mean (SD), ppm	1.68 (0.50)	1.71 (0.49)
ETCOc $\le$ 1.5 ppm, *n* (%)	521 (42.1)	489 (39.4)
1.5 $<$ ETCOc $<$ 2.5 ppm, *n* (%)	627 (50.7)	660 (53.2)
ETCOc $\ge$ 2.5 ppm, *n* (%)	89 (7.2)	92 (7.4)
Loss of follow-up during study period, *n* (%)	29 (2.3)	27 (2.2)

*GA* gestational age*, TcB* transcutaneous bilirubin*, ETCOc* end-tidal carbon monoxide-corrected*, IQR* interquartile range*, SD* standard deviation*, ppm* parts per million

### Primary outcome: the rate of phototherapy within seven days of life

PT within seven DOLs occurred in 281 (22.5%) of the 1248 infants in the control group and in 237 (18.9%) of the 1252 infants in the intervention group according to the ITT analysis [rate difference, − 3.43; 95% confidence interval (CI), − 4.67 to − 0.004; relative risk ratio (RR), 0.85; 95% CI, 0.73 to 0.98; *P* = 0.02] (Table 2). No significant interaction with sex or maternal blood group was observed.

**Table 2:** Comparison of outcomes between intervention and control groups

Measures	Control^a^ group	Intervention group	Group difference (95% CI)	Unadjusted analysis	Adjusted analysis	
				Rate/mean difference (95% CI)	Parameter estimate (95% CI)	*P* value
Primary outcome						
The rate of the first PT within 7 DOLs, *n* (%)	281 (22.5)	237 (18.9)	− 3.59 (− 6.84, − 0.33)	− 3.43 (− 4.67, − 0.004)	**0.85**^**b**^** (0.73, 0.98)**	**0.02**
Secondary outcomes						
Postnatal hours at first PT, median (IQR), h	68.7 (58.7)	58.1 (58.3)	− 5.90 (− 12.18, 0.00)	− 4.98 (− 12.0, 2.05)	− 4.98^c^ (− 12.01, 2.05)	0.17
TSB before PT, mean (SD), $\mu$ mol/L	206.5 (56.7)	203.4 (58.8)	− 3.11 (− 13.6, 7.34)	− 5.03 (− 13.2, 3.17)	− 1.27^c^ (− 11.49, 8.94)	0.81
TSB before PT $>$ 307.8 $\mu$ mol/L, *n* (%)	14 (5.6)	13 (5.8)	0.16 (− 0.05, 0.04)	0.04 (− 0.76, 0.84)	1.04^c^ (0.47, 2.29)	0.92
ETCOc before PT, median (IQR), ppm	1.9 (0.8)	2.1 (0.8)	0.29 (0.10, 0.50)	0.28^c^ (0.10, 0.46)	**0.28**^**c**^** (0.10, 0.46)**	** < 0.01**
PT duration, median (IQR), h	10 (14.8)	11.8 (16.6)	− 0.49 (− 1.00, 0.50)	1.07^c^ (− 1.68, 3.82)	1.07^c^ (− 1.68, 3.82)	0.45

Significant estimates in bold. *95% CI* 95% confidence interval*, PT* phototherapy *TSB* total serum bilirubin*, DOL* days of life*, IQR* interquartile range*, SD* standard deviation

^a^Control group as the reference level. ^b^Relative risk ratio from log-binomial regression adjusted for gestational age, birth weight, maternal blood group (model failed to converge when additionally adjusted for sex). ^c^Parameter estimates (beta coefficient, or adjusted mean difference or adjusted rate difference) from regressions adjusted for gestational age, birth weight, sex, maternal blood group

### Secondary outcomes

The postnatal age at first PT and the TSB before PT were comparable between the two groups. The ETCOc before PT was significantly greater in the intervention group ($\beta$ estimate = 0.28; 95% CI, 0.10 to 0.46; *P* $<$ 0.01). ETCOc-adjusted risk assessment to establish the PT threshold did not lead to significantly different proportions of severe hyperbilirubinemia (defined as TSB $>$ 307.8 $\mu$mol/L) or total duration PT (Table 2).

## Discussion

In this trial, incorporating ETCOc-adjusted risk assessment resulted in a significant decrease in the rate of PT within seven DOLs in 2500 near-term and term neonates. It did not increase the risk of severe hyperbilirubinemia, and no acute bilirubin encephalopathy was reported.

It is crucial to assess risk factors for developing NHB thoroughly as the threshold for PT initiation should be lower in neonates with an on-going hemolysis or a higher rate of bilirubin production. A delicate balance is needed to weigh the need for PT against risk assessment in terms of the pretest probability. Endogenously produced CO has been used as a noninvasive indicator of heme degradation [22]. It provides a quantifiable index of bilirubin production and offers valuable insights into the metabolism of bilirubin with clear clinical advantages [23]. Thus, ETCOc measurement, as an easy and fast test, can play an important role in ensuring an early postnatal risk assessment for hemolysis and bilirubin production during well-baby nursery stay and after well-baby nursery discharge.

Both the AAP and Chinese guidelines state that the ETCOc may be helpful in quantifying hemolysis [5, 6, 21], but current data are mainly observational-based, and the scarcity of evidence has limited its application. Previous studies have reported a link between ETCOc and TSB [8, 24, 25] and the potential of using ETCOc to manage hemolytic diseases [14–16, 25–27] and PT [16–18, 28]. In an observational diagnostic study comparing ETCOc with a direct antiglobulin test (DAT), Elsaie et al*.* reported that the use of ETCOc led to different management strategies in 10% of infants and led to fewer PTs by accurately identifying hemolyzing infants [14]. Although previous studies have reported an incremental relationship between TcB/TSB and ETCOc [8, 24, 25], the predictive performance of ETCOc varies. This might be due to differences in the study population, outcome definitions, and ETCOc thresholds. The current study is a large sample-size randomized clinical trial (RCT) that supports previous observational data and demonstrated that ETCOc-adjusted risk assessment for NHB leads to a significantly lower rate of PT in the early postnatal period.

In our study, we observed a 4% reduction in the first PT rate during the first seven days of life. This reduction is clinically significant given the large population base and the high incidence of jaundice in the Chinese population. This can possibly mitigate increasing health resource utilization and cost related to the NHB. The TSB levels before PT were comparable between the two groups. This is likely because TSB reflects a combination of bilirubin production, metabolism, and excretion. However, ETCOc levels were  elevated in the intervention group, but the difference was deemed not clinically significant. No increased risk of severe hyperbilirubinemia or acute bilirubin encephalopathy was reported during the trial. The ETCOc-adjusted approach demonstrated comparable clinical outcomes while significantly reducing phototherapy utilization within seven DOLs. Although ETCOc-adjusted thresholds effectively reduce phototherapy utilization, the current higher cost of ETCOc monitoring than conventional bilirubin testing (TcB/TSB) may limit its immediate adoption in resource-constrained settings. Nevertheless, these higher costs may be offset by reducing unnecessary phototherapy and associated healthcare utilization. Future cost-effectiveness analyses are needed to evaluate the long-term economic impact of this strategy across diverse healthcare systems.

To date, no established recommendations have been made with respect to settings, appropriate timing of measurements, and optimal thresholds of ETCOc. The results of this trial substantiate that ETCOc can be an optimal quantifiable parameter to identify hemolysis and can be integrated into the NHB risk category assessment, particularly during the early postnatal period ($\le$ 72 hours of birth) and for populations who are at a higher risk of hemolysis (e.g., present with a higher TcB/TSB shortly after birth). To our knowledge, this is so far the first large randomized control trial to evaluate the use of ETCOc to adjust NHB risk categories and PT threshold. There are currently no well-established ETCOc thresholds for identifying hemolysis although previous studies reported some single values on the basis of specific population distributions [16, 23, 29]. Therefore, we opted to use the threshold of 1.5 ppm to discriminate hemolytic risk, aligning with the algorithm suggested by Bhutani et al*.*[8] when we conceptualized this trial in 2021.

This study had several limitations. First, there may be possible unadjusted confounding effects, e.g., a history of inadequate feeding during well-baby nurseries, cephalohematoma, or significant bruising after birth. However, with a randomized controlled study design, this study was able to minimize biases related to baseline conditions. Second, this was a single-center RCT conducted among Chinese neonates in an area with a high incidence of neonatal hemolytic diseases, e.g., G6PD deficiency [30, 31]. The lack of ethnic and geographical diversity may limit its generalizability, and further studies in more diverse populations are warranted. We purposefully chose to perform a single-center trial for three main reasons: (1) as one of the largest delivery centers in the region with more than 20,000 annual deliveries, we should not encounter many difficulties in recruiting 2500 infants; (2) participants recruited from the well-baby nursery would be representative of the local population; and (3) we have adequate manpower and power dynamics to ensure the quality of the study at a single site. As the RCT to examine the role of ETCOc risk adjustment in NHB management and with a much larger sample size than did previous studies, the results of this study are likely applicable to other regions. Multicenter validation of the effect of the ETCOc-adjusted threshold remains essential. Third, we used the 2014 Chinese expert consensus on NHB management [21] in this study, which was in line with the 2004 AAP guidelines [5], because the 2022 AAP guidelines [6] and the 2025 Chinese expert consensus [32] had not yet been published at the time of this trial. Data from this trial could provide the basis for further clinical studies to understand the effect of ETCOc risk adjustment in the context of newer guidelines. Finally, since we chose to recruit infants with TcB $>$ 40th percentile, our study was not able to examine ETCOc as a general screening tool. To further explore clinical scenarios for ETCOc application, we are conducting a multicenter study in China recruiting all eligible neonates in well-baby nurseries with no prespecific threshold of TcB/TSB as a inclusion criterion.

In conclusion, among near-term and term Chinese neonates who had a TcB $>$ 40th percentile within 72 hours after birth, the use of ETCOc to adjust the NHB risk category significantly decreased the rate of PT in the first seven DOLs. The use of ETCOc did not increase the risk of subsequent severe hyperbilirubinemia.

## Supplementary Information

Below is the link to the electronic supplementary material.

Supplementary file1 (DOCX 88 KB)

## Data Availability

The datasets generated during and/or analyzed during the current study are available from the corresponding author on reasonable request.

## References

[1] Woodgate P, Jardine LA. Neonatal jaundice: phototherapy. BMJ. Clin Evid. 2015;2015:0319. PMC4440981 25998618

[2] Olusanya BO, Teeple S, Kassebaum NJ. The contribution of neonatal jaundice to global child mortality: findings from the GBD 2016 study. Pediatrics. 2018;141:e20171471. 29305393 10.1542/peds.2017-1471

[3] Bhatt P, Umscheid J, Ayensu M, Parmar N, Vasudeva R, Donda K, et al. Trends and resource utilization for neonatal jaundice hospitalizations in the United States. Hosp Pediatr. 2022;12:392–9. 35342924 10.1542/hpeds.2021-006269

[4] Khan AW, Bhatt P, Yagnik PY, Ayensu M, Adjetey NA, Agyekum AA, et al. Trends in hospitalization for neonatal jaundice and kernicterus in the United States, 2006–2017. Pediatrics. 2021;147:744–5.

[5] American Academy of Pediatrics Subcommittee on Hyperbilirubinemia. Management of hyperbilirubinemia in the newborn infant 35 or more weeks of gestation. Pediatrics. 2004;114:297–316. 15231951 10.1542/peds.114.1.297

[6] Kemper AR, Newman TB, Slaughter JL, Maisels MJ, Watchko JF, Downs SM, et al. Clinical practice guideline revision: management of hyperbilirubinemia in the newborn infant 35 or more weeks of gestation. Pediatrics. 2022;150:e2022058859. 35927462 10.1542/peds.2022-058859

[7] Castillo Cuadrado ME, Bhutani VK, Aby JL, Vreman HJ, Wong RJ, Stevenson DK. Evaluation of a new end-tidal carbon monoxide monitor from the bench to the bedside. Acta Paediatr. 2015;104:e279–82. 25640053 10.1111/apa.12938

[8] Bhutani VK, Srinivas S, Castillo Cuadrado ME, Aby JL, Wong RJ, Stevenson DK. Identification of neonatal haemolysis: an approach to predischarge management of neonatal hyperbilirubinemia. Acta Paediatr. 2016;105:e189–94. 26802319 10.1111/apa.13341

[9] Christensen RD, Lambert DK, Henry E, Yaish HM, Prchal JT. End-tidal carbon monoxide as an indicator of the hemolytic rate. Blood Cells Mol Dis. 2015;54:292–6. 25624169 10.1016/j.bcmd.2014.11.018

[10] Tidmarsh GF, Wong RJ, Stevenson DK. End-tidal carbon monoxide and hemolysis. J Perinatol. 2014;34:577–81. 24743136 10.1038/jp.2014.66

[11] Du L, Ma X, Shen X, Bao Y, Chen L, Bhutani VK. Neonatal hyperbilirubinemia management: clinical assessment of bilirubin production. Semin Perinatol. 2021;45:151351. 33308896 10.1016/j.semperi.2020.151351

[12] Christensen RD, Bahr TM, Pakdeeto S, Supapannachart S, Zhang H. Perinatal hemolytic disorders and identification using end tidal breath carbon monoxide. Curr Pediatr Rev. 2023;19:376–87. 36545740 10.2174/1573396319666221220095522

[13] Kaplan M, Herschel M, Hammerman C, Hoyer JD, Stevenson DK. Hyperbilirubinemia among African American, glucose-6-phosphate dehydrogenase-deficient neonates. Pediatrics. 2004;114:e213–9. 15286259 10.1542/peds.114.2.e213

[14] Elsaie AL, Taleb M, Nicosia A, Zangaladze A, Pease ME, Newton K, et al. Comparison of end-tidal carbon monoxide measurements with direct antiglobulin tests in the management of neonatal hyperbilirubinemia. J Perinatol. 2020;40:1513–7. 32203175 10.1038/s41372-020-0652-y

[15] Bhatia A, Chua MC, Dela Puerta R, Rajadurai VS. Noninvasive detection of hemolysis with ETCOc measurement in neonates at risk for significant hyperbilirubinemia. Neonatology. 2020;117:612–8. 32894848 10.1159/000509405 PMC7845425

[16] Bao Y, Zhu J, Ma L, Zhang H, Sun L, Xu C, et al. An end-tidal carbon monoxide nomogram for term and late-preterm Chinese newborns. J Pediatr. 2022;250:16–21.e3. 35835229 10.1016/j.jpeds.2022.07.003

[17] Bahr TM, Shakib JH, Stipelman CH, Kawamoto K, Lauer S, Christensen RD. Improvement initiative: end-tidal carbon monoxide measurement in newborns receiving phototherapy. J Pediatr. 2021;238:168–73.e2. 34260896 10.1016/j.jpeds.2021.07.008

[18] Zhan YL, Peng HB, Jin ZC, Su JF, Tan XY, Zhao L, et al. Higher ETCOc predicts longer phototherapy treatment in neonatal hyperbilirubinemia. Front Pediatr. 2023;11:1154350. 37114002 10.3389/fped.2023.1154350 PMC10126460

[19] Subspecialty Group of Neonatology, The Society of Pediatrics, Chinese Medical Association. The experts consensus on the management of neonatal hyperbilirubinemia. Zhonghua Er Ke Za Zhi. 2014;52:745–8. 25537539

[20] Bhutani VK, Johnson L, Sivieri EM. Predictive ability of a predischarge hour-specific serum bilirubin for subsequent significant hyperbilirubinemia in healthy term and near-term newborns. Pediatrics. 1999;103:6–14. 9917432 10.1542/peds.103.1.6

[21] Subspecialty Group of Neonatology, The Society of Pediatrics, Chinese Medical Association. The experts consensus on the management of neonatal hyperbilirubinemia. Zhonghua Er Ke Za Zhi. 2014;52:745–8. 25537539

[22] Maisels MJ, Pathak A, Nelson NM, Nathan DG, Smith CA. Endogenous production of carbon monoxide in normal and erythroblastotic newborn infants. J Clin Invest. 1971;50:1–8. 5543875 10.1172/JCI106463 PMC291888

[23] Christensen RD, Bahr TM, Wong RJ, Vreman HJ, Bhutani VK, Stevenson DK. A “gold standard” test for diagnosing and quantifying hemolysis in neonates and infants. J Perinatol. 2023;43:1541–7. 37468612 10.1038/s41372-023-01730-4

[24] Bhutani VK, Wong RJ, Vreman HJ, Stevenson DK. Bilirubin production and hour-specific bilirubin levels. J Perinatol. 2015;35:735–8. 25880796 10.1038/jp.2015.32

[25] Christensen RD, Malleske DT, Lambert DK, Baer VL, Prchal JT, Denson LE, et al. Measuring end-tidal carbon monoxide of jaundiced neonates in the birth hospital to identify those with hemolysis. Neonatology. 2016;109:1–5. 26394287 10.1159/000438482

[26] Kaplan M, Hoyer JD, Herschel M, Hammerman C, Stevenson DK. Glucose-6-phosphate dehydrogenase activity in term and near-term, male African American neonates. Clin Chim Acta. 2005;355:113–7. 15820485 10.1016/j.cccn.2004.12.008

[27] Cheng X, Lin B, Yang Y, Yu Y, Fu Y, Yang C. End-tidal carbon monoxide concentrations measured within 48 hours of birth predict hemolytic hyperbilirubinemia. J Perinatol. 2024;44:897–901. 38627593 10.1038/s41372-024-01967-7

[28] Stevenson DK, Fanaroff AA, Maisels MJ, Young BW, Wong RJ, Vreman HJ, et al. Prediction of hyperbilirubinemia in near-term and term infants. Pediatrics. 2001;108:31–9. 11433051 10.1542/peds.108.1.31

[29] Okuyama H, Yonetani M, Uetani Y, Nakamura H. End-tidal carbon monoxide is predictive for neonatal non-hemolytic hyperbilirubinemia. Pediatr Int. 2001;43:329–33. 11472573 10.1046/j.1442-200x.2001.01412.x

[30] Liu Z, Yu C, Li Q, Cai R, Qu Y, Wang W, et al. Chinese newborn screening for the incidence of G6PD deficiency and variant of G6PD gene from 2013 to 2017. Hum Mutat. 2020;41:212–21. 31489982 10.1002/humu.23911

[31] He Y, Zhang Y, Chen X, Wang Q, Ling L, Xu Y. Glucose-6-phosphate dehydrogenase deficiency in the Han Chinese population: molecular characterization and genotype-phenotype association throughout an activity distribution. Sci Rep. 2020;10:17106. 33051526 10.1038/s41598-020-74200-y PMC7555859

[32] Subspecialty Group of Neonatology, the Society of Pediatrics, Chinese Medical Association; Editorial Board, Chinese Journal of Pediatrics. Guidelines on the clinical management of neonatal hyperbilirubinemia (2025). Zhonghua Er Ke Za Zhi. 2025;63:338–350. 10.3760/cma.j.cn112140-20241231-00944 40090910

