# Supplementary material for: End-tidal CO corrected for ambient CO risk adjusted phototherapy threshold for the management of neonatal hyperbilirubinemia: a randomized clinical trial
Source: World J Pediatr. 2025 Aug 4;21(8):792–9. doi: 10.1007/s12519-025-00954-y (PMC12380970; doi:10.1007/s12519-025-00954-y)
Supplement: Supplementary file 1 — Supplementary file1 (DOCX 88 KB) [file 12519_2025_954_MOESM1_ESM.docx]

**SUPPLEMENTAL MATERIALS**

**
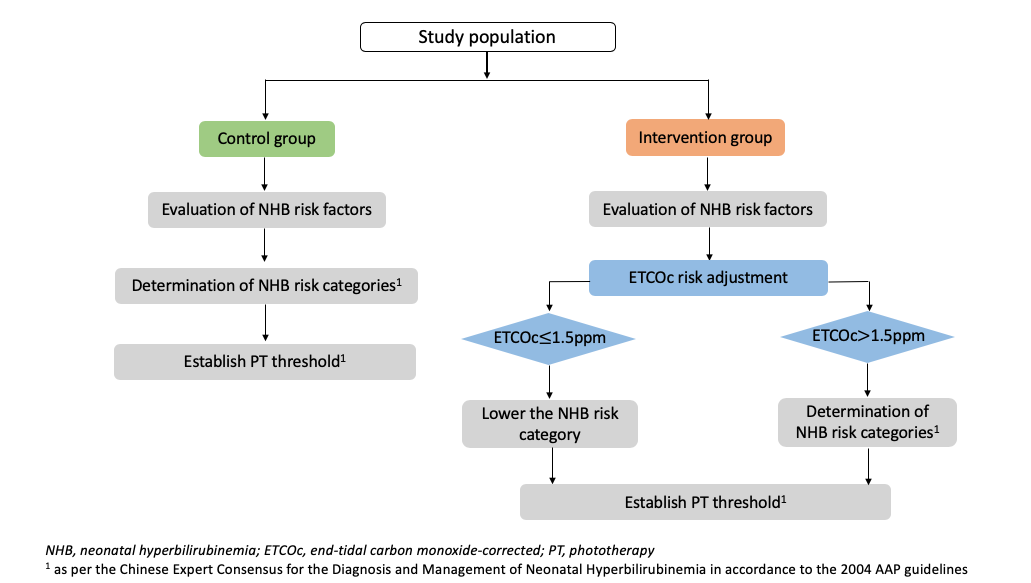
**

**Supplement Figure 1. Study procedure**

**Supplement Table 1. Comparison of participants who completed follow-up and who were lost of follow-up**

| **Characteristics** | **Participants, No. (%)** | |
| --- | --- | --- |
|  | **Participants who completed follow-up**  **(n = 2,444)** | **Participants who were lost to follow-up**  **(n = 56)** |
| Gestational age, median (IQR), weeks | 39.3 (1.43) | 39.9 (1.29) |
| Birth weight, mean (SD), g | 3,175 (393) | 3,220 (358) |
| Female sex, No. (%) | 1,181 (49.2) | 29 (51.8) |
| Mode of delivery, No. (%) |  |  |
| Vaginal delivery | 1,317 (54.9) | 35 (62.5) |
| Cesarean delivery | 915 (38.1) | 16 (28.6) |
| Forceps delivery | 169 (7.0) | 5 (8.9) |
| Maternal blood types, No. (%) |  |  |
| Non-type O | 1,440 (60.0) | 32 (40.0) |
| Type O | 961 (40.0) | 24 (42.9) |
| Age at recruitment, mean (SD), hours | 26.7 (13.6) | 25.3 (11.3) |
| TcB at recruitment, median (IQR), mg/dl | 6.7 (2.9) | 6.6 (2.0) |
| ETCOc at recruitment, mean (SD), ppm | 1.70 (0.49) | 1.59 (0.48) |
| ETCOc $\leq$ 1.5 ppm, No. % | 968 (40.6) | 28 (52.8) |
| 1.5 $<$ ETCOc $<$ 2.5 ppm, No. % | 1,241 (52.1) | 23 (43.4) |
| ETCOc $\geq$ 2.5 ppm, No. % | 173 (7.3) | 2 (3.8) |

*Abbreviation: GA, gestational age; TcB, transcutaneous bilirubin; ETCOc, end-tidal carbon monoxide-corrected; IQR, interquartile range; SD, standard deviation; ppm, parts per million*
